# Synthetic Lipopeptide Enhances Protective Immunity Against *Helicobacter pylori* Infection

**DOI:** 10.3389/fimmu.2019.01372

**Published:** 2019-06-14

**Authors:** Ruo-Yi Xue, Mu-fei Guo, Ling Guo, Chang Liu, Sun Li, Jiao Luo, Li Nie, Lu Ji, Cong-Jia Ma, Da-Qun Chen, Si Sun, Zhe Jin, Quan-Ming Zou, Hai-Bo Li

**Affiliations:** ^1^Department of Microbiology and Biochemical Pharmacy, National Engineering Research Center of Immunological Products, College of Pharmacy, Third Military Medical University, Chongqing, China; ^2^Chongqing Nankai Secondary School, Chongqing, China; ^3^Chongqing Technical Center for Drug Evaluation and Certification, Chongqing, China

**Keywords:** *Helicobacter pylori*, vaccine, synthetic lipopeptide, TLR2, HpaA

## Abstract

Over fifty percent of the people around the world is infected with *Helicobacter pylori* (*H. pylori*), which is the main cause of gastric diseases such as chronic gastritis and stomach cancer. *H. pylori* adhesin A (HpaA), which is a surface-located lipoprotein, is essential for bacterial colonization in the gastric mucosa. HpaA had been proposed to be a promising vaccine candidate against *H. pylori* infection. However, the effect of non-lipidated recombinant HpaA (rHpaA) to stimulate immune response was not very ideal, and the protective effect against *H. pylori* infection was also limited. Here, we hypothesized that low immunogenicity of rHpaA may attribute to lacking the immunostimulatory properties endowed by the lipid moiety. In this study, two novel lipopeptides, LP1 and LP2, which mimic the terminal structure of the native HpaA (nHpaA), were synthesized and TLR2 activation activity was confirmed *in vitro*. To investigate whether two novel lipopeptides could improve the protective effect of rHpaA against the infection of *H. pylori*, groups of mice were immunized either intramuscularly or intranasally with rHpaA together with LP1 or LP2. Compared with rHpaA alone, the bacterial colonization of the mice immunized with rHpaA plus LP2 via intranasal route was significantly decreased and the expression levels of serum IgG2a, IFN-γ, and IL-17 cytokines in spleen lymphocyte culture supernatant increased obviously, indicating that the enhanced protection of LP2 may be associated with elevated specific Th1 and Th17 responses. In conclusion, LP2 has been shown to improve the protective effect of rHpaA against *H. pylori* infection, which may be closely related to its ability in activating TLR2 by mimicking the terminal structure of nHpaA.

## Introduction

*Helicobacter pylori* (*H. pylori*) was identified as a category I human carcinogen by the International Agency for Research on Cancer in 1994. Infection with *H. pylori* was proved to be associated with peptic ulcer, chronic gastritis and gastric carcinoma. It is estimated that over half of the world's population is infected with *H. pylori* (1–3). Due to the growing antibiotic resistance, the clearance rate of *H. pylori* has fallen in a great portion of patients (4–6), and the treatment of *H. pylori* infection has become a challenge in recent years. Thus, vaccination may be one of the most promising means to control *H. pylori* infection (7).

In the pathogenesis of *H. pylori*, bacterial attachment to mucosal epithelial cells and gastric epithelial mucosa seems to be a crucial step (8, 9). Because of their important role in host adhesion and survival, membrane proteins are considered as potential targets for vaccine development. HpaA (*H. pylori* adhesin A), also described as neuraminyllactose-binding haemagglutinin (NLBH), can bind to various glycosylation components on the surface of gastric epithelial cells, which is essential for colonization of the bacteria to gastric mucosa (10, 11). It has previously been found that expression of HpaA is highly conserved in *H. pylori* isolates. (12). Genomic studies also showed that HpaA has no significant sequence homologies with other known proteins (13). Additionally, several studies had confirmed the immunogenicity and immunostimulatory effect of HpaA, while the expression of serum HpaA-specific antibodies was elevated in those infected with *H. pylori* (14, 15). HpaA had been considered as a promising *H. pylori* vaccine candidate antigen.

It was reported that the prophylactic immunization with non-lipidated recombinant HpaA (rHpaA) could induce protection against *H. pylori* infection in mice (16–19). However, the effect of stimulating human immune response was not satisfactory, and the protection against the infection of *H. pylori* was also limited (20, 21). However, native HpaA (nHpaA) from *H. pylori* is a surface-located lipoprotein, which may play an important role in activating innate and adaptive immune responses via TLR2-dependent signaling pathways (22–25). In addition, lipoprotein could activate macrophages and monocytes, which may promote immune response and play a role as an adjuvant (26–28). Based on these studies, we deduce that the lack of lipid modification on the rHpaA in *Escherichia coli* may be the main reason for its weak immunostimulatory and protective effects.

It had been reported that lipoproteins could be recognized by TLR2 mainly depending on their N-terminal lipid moiety (29, 30). Therefore, the capability of lipoproteins to activate TLR2 could be retained by mimicking the specific N-terminal structure. In this study, the lipid modification sites of nHpaA were predicted by bioinformatics. Accordingly, two novel lipopeptides, including LP1 and LP2, were synthesized. Mice were immunized with the mixture of synthetic lipopeptides and rHpaA through intramuscular or intranasal routes. The protection against the infection of *H. pylori* was evaluated and the mechanism was explored.

## Materials and Methods

### Ethics Statement

Animal maintenance and laboratory procedures were carried out according to the National Institutes of Health Guidelines for the Use of Experimental Animals and approved by the Medicine Animal Care Committee of the Third Military Medical University. Well-trained and skilled animal care personnel participated in the current study to minimize the suffering of animals. All procedures were performed under anesthesia with 1% sodium pentobarbital to alleviate pain. The health of the mice we monitored every 8–12 h and CO_2_ was used for euthanasia.

### Animals and Cell Lines

Specific-pathogen-free (SPF) female BALB/c mice aged 6–8 weeks old were purchased from the Experimental Animal Center of the Third Military Medical University, and were kept under pathogen-free conditions. HK-2 (human kidney 2) was purchased from the ATCC (American Type Culture Collection). HEK-Blue™-mTLR2 cells were purchased from InvivoGen (San Diego, CA, USA) and cells were grown in DMEM supplemented with 10% FBS, L-glutamine (2 mM), Normocin (100 μg/mL), penicillin (50 U/mL), streptomycin (50 g/mL) and passaged when reached 70% confluence. Cells were scraped and then resuspended in HEK-Blue™ Detection medium (InvivoGen, San Diego, CA, USA). The induction of SEAP was detected at 620 nm by a spectrophotometer.

### Reagents

**Figure 1:**
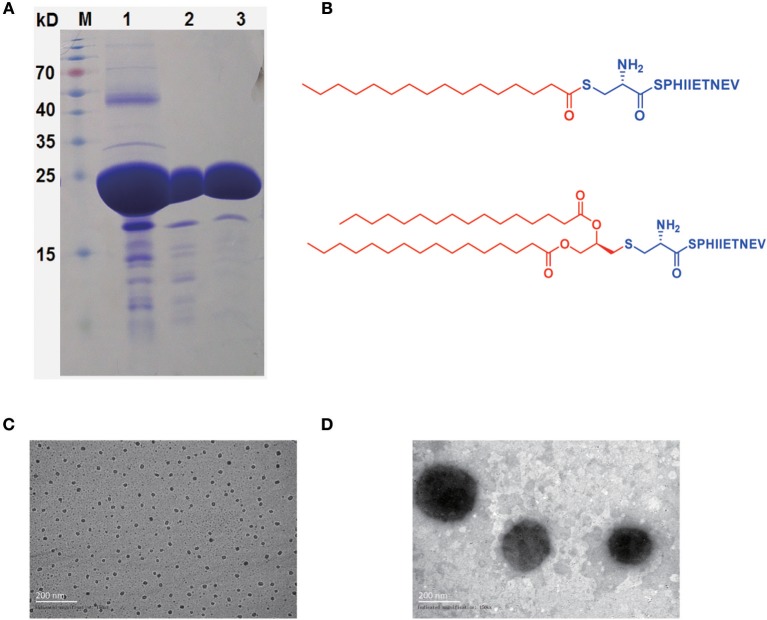
rHpaA expression and lipopeptides characterization. **(A)** SDS-PAGE. rHpaA was expressed in *E. coli*. rHpaA was firstly purified by affinity chromatography (lane 1) and then refined by ion exchange chromatography (lane 2). Size exclusion chromatography was used in the final step to polish the purification (lane 3). **(B)** Chemical structure of LP1 and LP2. Transmission electron microscopy (TEM) image of LP1 **(C)** and LP2 **(D)** micelles after dying with 2% phosphotungstic acid.

rHpaA was expressed in *E. coli* and purified as previously described (Figure 1A) (31, 32). *Helicobacter Pylori* Medium (HB8674) was purchased from Hopebio (Qingdao, Shandong, China). HRP conjugated goat anti-mouse IgG (ab97200), IgG1 (ab97240), IgG2a (ab97245), and IgA (ab97235) were the products of Abcam (Cambridge, MA, USA). Mouse IFN-γ (DKW12-2000-096), IL-17A (DKW12-2170-096), IL-4 (DKW12-2040-096) precoated ELISA kit were purchased from Dakewe (Shenzhen, Guangdong, China). Anti-mouse CD11c-FITC (117305), CD80-APC (104713), CD86-PerCP (105025) were the products of BioLegend (San Diego, MA, USA).

### Synthesis of Lipopeptides

LP1 and LP2 were synthesized with solid-phase methods as previously described (33). LP1 was obtained by S-acylation of the cysteine of the peptide (CSPHIIETNEV) with palmitoyl chloride, and then the lipopeptide is cleaved from the resin and purified. Fmoc-Pam_2_Cys-OH was introduced to the N terminus of the peptide (SPHIIETNEV) linked to the resin. The Fmoc group was subsequently cleaved. The molecular were detached from the resin and all protecting groups were deprotected to yield the lipopeptide LP2.

### Hemolysis and Cell Viability Assay

Hemolysis and cell viability assay was performed using methods described previously (34). Briefly, 0.5 mL of 2% solution of mice red blood cells was mixed with 0.5 mL of diluent containing 100, 200, 400, and 1.25 μg/mL of LP1 or LP2 and then incubated at 37°C for 3 h. The hemolytic activity was determined by measuring the absorbance of the supernatant at 570 nm. For cell viability assay, the trypsin-treated HK-2 cells were cultured with gradient dilutions of LP1 or LP2 at for 48 h. Afterward, 20 μL of a MTT solution (5 mg/mL) was added to each well and incubated for 4 h at 37°C. Subsequently, the mixture was removed from the well and 100 μL DMSO was added. The optical absorption was detected at a wavelength of 570 nm. Percentage of surviving cells = Mean optical absorption of cells exposed to the lipopeptide/Mean optical absorption of control cells × 100.

### Transmission Electron Microscopy (TEM)

The morphology of the synthetic lipopeptides was investigated by TEM negative staining technique. LP1 or LP2 at 100 μg/mL was deposited onto a copper grid and negative stained with 2% phosphotungstic acid for 30 s. After removal of excess of the solution and kept dried for at least 6 h, TEM images were taken by a JEOL JEM-1230 (JEOL Ltd, Tokyo, Japan) (35).

### TLR2 Signaling Assay

The sample was added to a flat-bottom 96-well plate (20 μL per well) and an equal amount of endotoxin-free water was used as a negative control. Followed by HEK-Blue™ mTLR2 cells were resuspended in the pre-warm HEK-Blue™ Detection, cell suspension (180 μL, 5 ×10^4^ cells) was added to the sample and then incubated at 37°C for 14 h. Samples were analyzed for SEAP activity after which OD was measured in a spectrophotometer at 620 nm (36).

### Generation of BMDCs and FACS Assay

Mouse bone marrow cells were isolated from tibia and femurs, and then were cultured in RPMI 1640 medium containing 10% FCS, recombinant murine GM-CSF (6 ng/mL) and IL-4 (20 ng/mL) at 37°C, 5% CO_2_. The non-adherent cells were removed on day 5 and adherent cells were cultured in a fresh complete medium for another 2 days. BMDCs were harvested and stimulated with LP1, LP2 or PBS for 48 h. After three washes, the cells were incubated with FITC-αCD11c, APC-αCD80, and PerCP-αCD86 in the dark for 30 min at 4°C. Flow cytometry data were acquired with a FACS Canto II (BD Biosciences) (37).

### Animal Immunization

For the different delivery routes, mice were divided randomly into two sections. In the first section, mice were intranasally immunized 5 times in 4 weeks with PBS, rHpaA alone, or rHpaA plus one of the two synthetic lipopeptides (2 μg/mouse). The final amount of rHpaA for each intranasal immunization was 10 μg in 20 μL PBS. In the second section, the mice were immunized intramuscularly three times at intervals of 2 weeks with 50 μg of antigen in the same groups to the first section. On day 43, sera and intestinal lavage fluid were collected for further determination.

### *H. pylori* Challenge and Quantification

Two weeks following the last immunization, mice were orally challenged with 10^9^ BALB/c mouse-adapted *H. pylori* four times for 4 days. At 2-week post-challenge, the number of *H. pylori* colonization was quantified by real-time PCR (38). The following primers were used: Forward, 5′-TTTGTTAGAGAAGATAATGACGGTATCTAAC-3′, Reverse, 5′-CATAGGATTTCACACCTGACTGACTATC-3′, Probe, 5′-FAM-CGTGCCAGCAGCCGCGGT-TAMRA-3′. This assay was performed in a Bio-Rad iQ5 multicolor real-time PCR thermocycler (39).

### Antibody Production Assay

Antibody levels were measured by standard indirect ELISA as previously described (40). Briefly, 96-well flat bottom microtiter plates were coated with rHpaA antigens in PBS and incubated overnight at 4°C. After blocking with 5% BSA in PBST, the 1:1,000 pre-diluted sera samples (intramuscularly) and the 1:50 pre-diluted sera samples (intranasally) were added to 96-well microtiter plates followed 1:1 gradient dilution and incubated for 1 h at 37°C. HRP-conjugated goat anti-mouse IgG was then used as a secondary antibody to determine antigen-specific IgG level. Each well was added 100 μL TMB substrate solution and then was kept in darkness for 20 min. The reaction was finally quenched by stop solution (2M sulfuric acid). The optical density (OD) was measured at 450 nm in a micro-plate reader (Bio-Rad). As described above, the levels of specific IgG1 and IgG2a in the serum were also measured by ELISA.

### Cytokines Assay

Splenocytes were isolated from mice and re-stimulated with the antigen for 48 h. Cytokines IFN-γ, IL-4, and IL-17A in coculture supernatants, which were derived from Th1/Th2/Th17 cells, were detected by ELISA kit. Absorbance at 450 nm was measured in a microplate reader.

### *In vitro* Adhesion Assay With AGS Cells

The *in vitro* adhesion assay was based on the method as previously described (41, 42). Briefly, *H. pylori* strain B0 were suspended in DMEM/F12 to an OD_600nm_ of 0.7 (i.e., 10^8^ CFU/ml), and then left untreated or treated with antiserum (1:100 dilution) at 4°C for 1 h. AGS cells were incubated with pretreated bacterial suspension at an MOI of 100 for each species. After 2 h of incubation, the cells were washed with PBS three times in order to remove unbound bacteria. The colonization of *H. pylori* attached to AGS cells was quantified by real-time PCR as described in section *H. pylori* Challenge and Quantification.

### Statistical Analysis

All statistical analysis were conducted by the SPSS 21.0 statistical package. Data were expressed as means ± standard deviation (SD). One-way ANOVA was used to analyze inter-group differences, and differences with a *p*-value ≤ 0.05 were considered statistically significant., Bonferroni test were further performed if inter-group differences were statistically significant, with the significant level α' = α/k (k is the number of comparison times).

## Results

### Synthesis and Characterization of LP1 and LP2

According to bioinformatics analysis using the DOLOP (http://www.mrc-lmb.cam.ac.uk/genomes/dolop) and UniProt (https://www.uniprot.org) database, nHpaA has two lipidative forms, monopalmitoylation (Pam) and dipalmitoylation (Pam2). The peptide containing 10 amino acids (CSPHIIETNEV), derived from the N terminus of the nHpaA, was selected for chemical coupling with Pam and Pam2, respectively, resulting in two novel lipopeptides (Figure 1B). The purity of the synthesized LP1 was 95.58% and of LP2 was 95.14% as determined by HPLC. The structures of LP1 and LP2 were further confirmed by mass spectrometry (Figures S1, S2.)

Given that LP1 and LP2 were amphiphilic molecules, as shown in Figures 1C,D, they were able to self-assemble into micelles in an aqueous environment. The resultant LP1 micelles had a mean diameter of about 20–30 nm (Figure 1C), and LP2 micelles were approximately ten times larger than LP1 micelles, with a diameter about 200 nm (Figure 1D). LP1 and LP2 micelles exhibited sizes in the range of microorganisms, which may be better recognized by antigen-presenting cells, thereby possibly enhanced the immune response against co-administered antigens.

### *In vitro* Safety of LP1 and LP2

**Figure 2:**
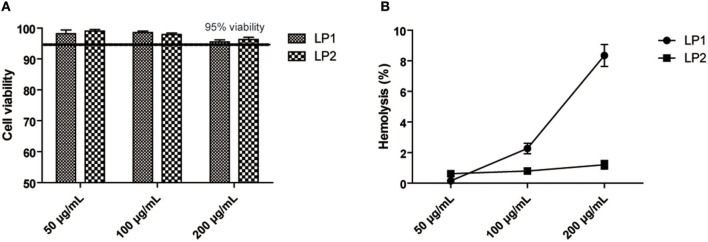
Cell viability and Hemolysis assay. **(A)** HK-2 cells were incubated with LP1 and LP2 for 48 h, respectively, and then cell viability was quantified with an MTT assay (data are expressed as mean ± S.D., *n* = 3). **(B)** Hemolytic effects were investigated by incubating erythrocytes with the diluent containing 200, 100, 50 μg/mL of two synthetic lipopeptides in saline (data are expressed as mean ± S.D., *n* = 3).

To evaluate the safety of LP1 and LP2 *in vitro*, the cytotoxicity of LP1 and LP2 was evaluated on HK-2 cell lines using the MTT colorimetric assay. As shown in Figure 2A, all doses tested (50–200 μg/ml) showed almost no HK-2 cells toxicity. Exposition of mice red blood cells to LP2 (50–200 μg/ml) did not induce significant hemolysis *in vitro*. LP1 caused <3% hemolysis at 100 μg/mL (Figure 2B). The above results indicated that synthetic lipopeptides were safe *in vitro* at concentrations below 100 μg/ml and could be further tested *in vivo*.

### LP1 and LP2 Activate TLR2 and Promote BMDCs Maturation

**Figure 3:**
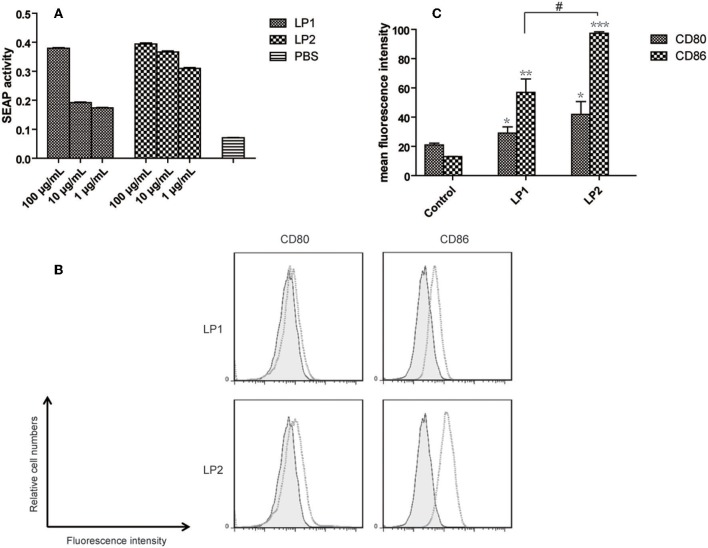
TLR2 signaling assay and expression of maturation surface markers in BMDCs treated with LP1 and LP2. **(A)** The effect of different lipopeptides treatment on secreted embryonic alkaline phophatase (SEAP) release from HEK-Blue™ mTLR2 cells. Data are expressed as mean ± S.D., *n* = 3. BMDCs were incubated with PBS, LP1, or LP2 for 48 h and analyzed for surface expression of CD80 and CD86. **(B)** A representative set of flow cytometry histograms. Black line: cells treated with PBS. **(C)** Normalized expression level of maturation markers. Data are expressed as mean ± S.D., *n* = 3. *P < 0.05, **P < 0.01, ***P < 0.001 compared with PBS control; ^#^*P* < 0.05.

To confirm whether the two synthesized lipopeptides retained the TLR2-stimulating activity of nHpaA, HEK293-TLR2 cells were used for luciferase reporter assays. As shown in Figure 3A, both LP1 and LP2 significantly enhanced the dose-dependent NF-kB activation in HEK293-TLR2 cells. Compared with LP1, LP2 exhibited significantly stronger TLR2 stimulatory activity.

To determine whether the synthetic lipopeptiedes has a direct effect on dendritic cells, BMDCs were stimulated with PBS, LP1 or LP2 for 2 days. FACS results showed that the levels of the costimulatory molecules (CD86 and CD80) expression were significantly elevated in LP1 and LP2 stimulation group compared with PBS group (Figure 3B). LP2 showed more potent stimulating effect (Figure 3C). Together, these data demonstrated that LP1 and LP2 had an ability to induce BMDCs maturation possibly through TLR2 signal pathway.

### LP2 Enhanced rHpaA-Induced Protective Effect Against *H. pylori* Infection via Intranasal Administration

**Figure 4:**
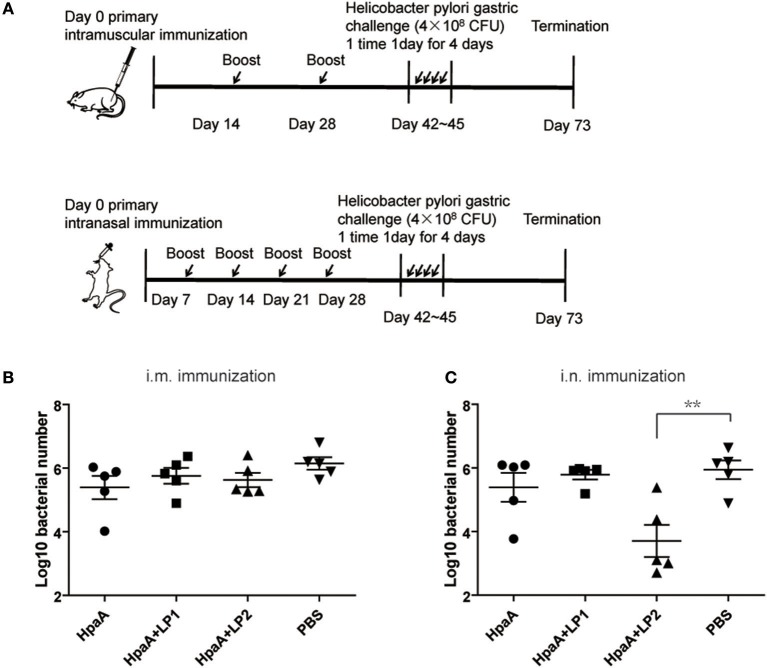
Colonization of *H. pylori* in stomachs of mice prophylactically immunized with rHpaA plus LP1 or LP2. **(A)** Timeline representation of immunization schedule and experimental procedures. BALB/c mice were intramuscularly or intranasally immunized with rHpaA or together with one of the two lipopeptides. Controls were treated with PBS. Two weeks after final vaccination, mice were challenged orally four times with *H. pylori* B0. Four weeks post challenge, levels of gastric *H. pylori* colonization via intramuscular **(B)** and intranasal **(C)** administration were determined by real-time quantitative PCR. Data are expressed as mean ± S.D., *n* = 5. **P < 0.01 compared with PBS control.

Since LP1 and LP2 could help to induce BMDCs maturation and showed no significant toxicity *in vitro*, we further investigated whether vaccinated with rHpaA and the synthetic lipopeptides could provide more potent protection against *H. pylori*. BALB/c mice were intramuscularly or intranasally immunized with rHpaA plus LP1 or LP2, and then orally challenged with *H. pylori* strain B0. Bacteria colonization in mice stomachs were determined by real-time PCR (Figure 4A). There was no protective effect was observed in the intramuscular immunization groups, whether rHpaA alone or in combination with one of the two lipopeptides (Figure 4B). However, the number of copies of *H. pylori* in the group intranasally immunized with rHpaA plus LP2 was significantly lower than that in the PBS group. No significant difference in bacterial load was observed in other intranasal immunization groups (Figure 4C). Those results indicated that, of the two lipopeptides, LP2 enhanced rHpaA-mediated protection against *H. pylori* when combined with rHpaA via intranasal administration.

### LP2 Elevated rHpaA Specific Serum IgG2a Response

**Figure 5:**
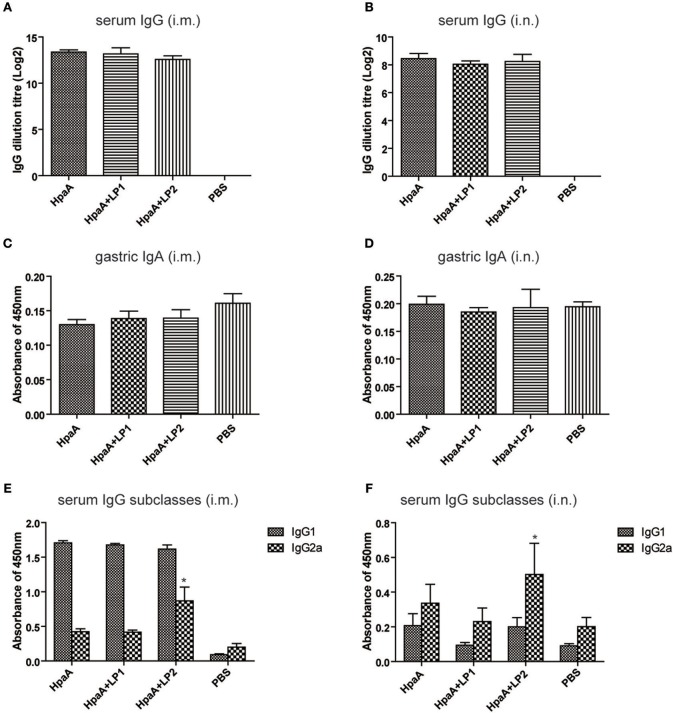
rHpaA-specific antibody profile of mice immunized with rHpaA plus LP1 or LP2. BALB/c mice were immunized intramuscularly or intranasally with rHpaA alone or rHpaA plus one of the two lipopeptides. The serum and intestinal lavage fluid was collected at two weeks after the last immunization. **(A,B)** rHpaA -specific serum IgG antibody titers were measured by ELISA. **(C,D)** Secretory IgA level in the intestinal lavage fluid. **(E,F)** The level of specific IgG1 and IgG2a against rHpaA in serum samples were tested by ELISA. Data are expressed as mean ± S.D., *n* = 5. *P < 0.05, compared with rHpaA alone group.

In order to investigate whether the synthetic lipopeptide could enhance rHpaA-specific humoral immune response via TLR2 activation, mice were immunized intranasally or intramuscularly with rHpaA plus LP1 or LP2, and the level of serum specific IgG were measured by ELISA. rHpaA in combination with the lipopeptides elicited significant serum specific IgG antibody response compared with PBS group (Figures 5A,B). Moreover, intramuscular immunization induced more robust IgG level than the same vaccine given intranasally. However, there was no significant difference in titer between rHpaA alone and plus one of the two lipopeptides whether administered intramuscularly or intranasally. In addition, two routes of administration failed to elicit specific IgA response (Figures 5C,D). Those results suggested that LP1 or LP2 could not elevate rHpaA specific systemic or local antibody response. IgG1 and IgG2a as markers for Th2 and Th1 responses respectively, and rHpaA-specific IgG1 and IgG2a levels were also determined. Compared with rHpaA alone group, both intranasal and intramuscular immunization plus LP2 resulted in elevated levels of serum IgG2a (Figures 5E,F). The results showed that enhanced rHpaA specific Th1 response were induced by LP2, which may be associated with improved protective efficacy against *H. pylori* infection.

### LP2 Induced Specific Th1 and Th17 Response When Intranasally Administrated With rHpaA

**Figure 6:**
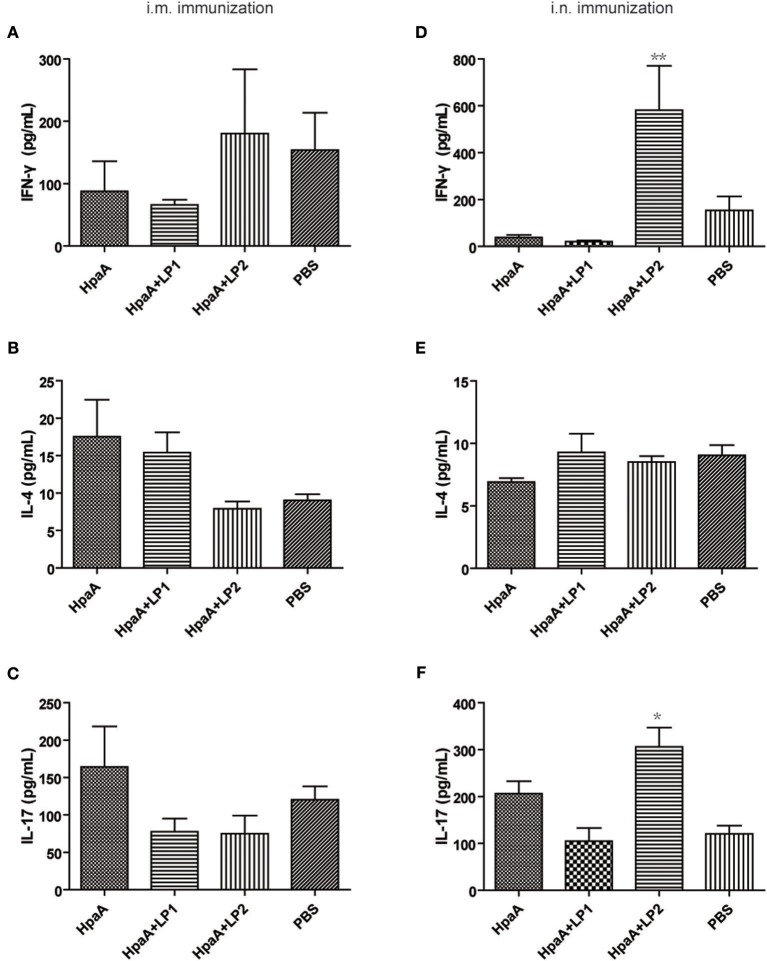
Cytokines production after stimulation of splenic lymphocytes with rHpaA. Mice were immunized intranasally or intramuscularly with rHpaA, rHpaA plus LP1, or rHpaA plus LP2, respectively. Six week after final immunization, splenic lymphocytes from vaccinated group were isolated and stimulated with 10 μg/mL rHpaA for 48 h. ELISA assays were used to measure the accumulation of IFN-γ **(A,D)**, IL-4 **(B,E)**, and IL-17 **(C,F)** in the supernatants of culture. Data are expressed as mean ± S.D., *n* = 5. **P < 0.01, *P < 0.05, compared with rHpaA alone group.

To further investigate the protective mechanism of LP2 assisted rHpaA against *H. pylori*, splenic lymphocytes were restimulated with the antigen and the CD4+ T cell response was measured by the expression levels of IFN-γ, IL-4, and IL-17 in supernatants, respectively. As shown in Figures 6A–C, there was no significant difference in the expression of IFN-γ, IL-4, and IL-17 among the intramuscularly immunized groups. When intranasally administrated in combination with rHpaA, LP2 can significantly promote IFN-γ/IL-17 levels Figures 6D–F), which suggested that LP2 could effectively elevate rHpaA specific Th1/Th17 response. Many previous studies have also emphasized the crucial role of vaccine-mediated Th1 and Th17 immune responses in protection against *H. pylori* infection (43, 44).

### LP2- or Peptide-Induced Specific Response Was not Involved in the Protection

**Figure 7:**
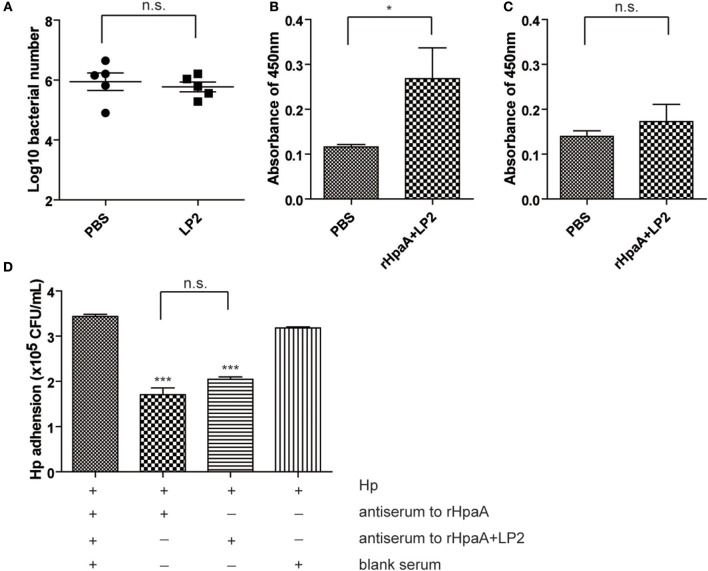
BALB/c mice were intranasally immunized with LP2 alone or PBS. Two weeks after final vaccination, mice were challenged orally four times with *H. pylori* B0. Four weeks post challenge, levels of gastric *H. pylori* colonization were determined by real-time quantitative PCR **(A)**. LP2-specific **(B)** or peptide-specific **(C)** antibody profile of mice intranasally immunized with rHpaA plus LP2. Data are expressed as mean ± S.D., *n* = 5. *P < 0.05. Adhesion of the *H. pylori* strain B0 to AGS cells alone or in combination with antiserum to rHpaA or antiserum to rHpaA plus LP2 **(D)**. The numbers of CFU/ml were determined by real-time PCR using the TaqMan method. The data shown are representative of three independent experiments with triplicate samples. Data are expressed as mean ± S.D., *n* = 3. ***P < 0.001, compared with blank serum group.

Given that intranasal immunization LP2 alone failed to confer protection against *H. pylori* infection (Figure 7A), we investigated whether the peptide moiety (SPHIIETNEV) of LP2 or LP2 specific response was involved in the protection. LP2 and peptide specific IgG titers in the sera of mice immunized with rHpaA+LP2 were measured. Intranasal immunization with HpaA+LP2 induced low levels of anti-LP2 IgG antibodies (Figure 7B), but anti-peptide antibody response was not observed (Figure 7C). In order to characterize the functional role of the anti-LP2 IgG antibodies, antiserums from mice intranasally immunized with rHpaA alone or plus LP2 were used to determine their effects on *H. pylori* adhesion. As shown in Figure 7D, both antiserums could significantly reduce binding of the *H. pylori* to AGS cells. However, there was no difference in the inhibitory effect on adhesion between the two antiserums. The above result suggested that anti-LP2 IgG were not involved in the protection. Further, splenic lymphocytes collected from mice immunized with rHpaA+LP2 were restimulated with LP2 or the peptide, and the levels of IFN-γ, IL-4, and IL-17 production in supernatants were measured, respectively. There was no significant difference in IFN-γ, IL-4, or IL-17 levels between rHpaA+LP2 group and PBS group, suggesting that rHpaA+LP2 could not elicit LP2- or peptide-specific CD4+ T cell response (Figure S3). Together, we concluded that LP2 or peptide moiety specific response was not involved in the protection in the current study.

## Discussion

*H. pylori* infection causes gastritis, gastric adenocarcinoma, gastroduodenal ulcers and mucosa-associated tissue lymphoma. With the increasing resistance to antibiotics, the success rate of standard therapy for *H. pylori* infection has recently declined to low levels, which highlights the need for a vaccine to control the pathogen. Some subunit vaccines have been studied as candidates for *H. pylori* vaccine, including single (UreB, VacA, CagA, NAP,) and multi-component vaccines (45). Also, HpaA has been found to be a promising vaccine candidate, which is essential for colonization and establishment of infection. In the previous studies, the antigenicity and immunogenicity of rHpaA were confirmed and intragastric immunization with rHpaA could confer protection against *H. pylori* in mice (14, 15, 17).

However, rHpaA-induced protective efficacy was usually limited or partially effective. Though intragastricly or intranasally immunized with rHpaA plus cholera toxins induced specific antibodies, bacterial colonization was slightly reduced compared with the control group. Our previous study also showed that intranasal immunization with rHpaA adjuvanted with CpG failed to confer protection against *H. pylori* (46). Compared with nHpaA, the absence of lipid modification on rHpaA may relate to the weak immunostimulatory and protective effects. In the current study, to simulate lipidative N terminus structure of nHpaA, two novel lipopeptides, LP1 and LP2, were synthesized with solid-phase methods. When the concentrations were lower than 100 μg/mL, LP1 and LP2 showed satisfactory hemolytic tolerance and no significant toxicity to HK-2 cells. TEM results indicated that LP1 and LP2 were able to self-assemble into micelles with diameters in the range of microorganisms, and thus they could be easily recognized by DC. In addition, LP1 and LP2 can activate TLR2 *in vitro* in a dose-dependent manner, indicating that the synthetic lipopeptides that mimic the lipid moiety of nHpaA retained TLR2-stimulating activity. Many studies indicated that co-administration of TLR2 agonists with antigen was able to induce DC maturation, which led to the up-regulation of costimulatory molecules (e.g., CD80, CD86) (28), and promote lymphocytes maturation and activation. In our study, LP1 and LP2 were also able to significantly promote DC maturation, which may be associated with their ability to activate TLR2.

One essential question remains: what type of immunity is key to eliminate *H. pylori* infection? Recent studies suggested that high levels of IgG antibodies may not be necessary for vaccine protection (47). In this study, no significant difference in serum IgG antibody levels was observed, regardless of the combination with LP1 or LP2. Given earlier studies indicating elevated levels of IgA levels were related to lower bacterial density, which suggested that sIgA might has protective role against *H. pylori* infection (48, 49), we also evaluated the level of intestinal IgA. However, levels of specific IgA did not significantly increase after immunization with rHpaA alone or plus the lipopeptides. Studies had shown that vaccines against *H. pylori* could be successfully inoculated in mice with deficiencies of the antibody or Th2 response, confirming that specific cellular immunity, instead of humoral immunity, was essential to eradicate *H pylori* (50), especially Th1 and Th17 cellular immune responses (51–54). In our study, as indicated by the increase of IgG2a level and IFN-γ/IL-17 cytokine concentrations, Th1 and Th17 response were elicited in mice intranasally immunized with rHpaA plus LP2. We hypothesized that LP2 acquired the ability to activate TLR2 by mimicking the terminal structure of nHpaA, and elevated rHpaA specific Th1 and Th17 response via intranasal administration, which resulted in the enhanced protection of rHpaA against *H. pylori* infection.

Though both LP1 and LP2 were synthetic lipopeptides based on the structure of nHpaA, there was significant difference in assisting rHpaA to provide protection against *H. pylori* infection. LP2, other than LP1, induced rHpaA specific cellular immunity and reduced bacterial colonization in mice. We speculated that the possible reason was that LP2 was more capable of activating TLR2 and promoting DC maturation. In addition, the vaccination route is also important for protective immunity (55). Previous studies have also reported that intranasal administration could provide more pronounced protective effect against *H. pylori* compared with intramuscular immunization, which was consistent with our results. The possible reason is that the upper respiratory tract (nasal-associated lymphoid tissue, NALT) is rich in APCs, especially mucosal DCs which express TLR on the surfaces (56, 57). Synthetic lipopeptides binded to TLR2 may promote DCs to uptake antigens and then present them to Th cells to induce a stronger immune response.

In this study, the synthetic lipopeptide LP2 was proved to enhance the protective effect of rHpaA against *H. pylori* infection via intranasal immunization, which might be mediated by specific Th1 and Th17 responses. In future studies, the mechanisms which were involved in the immune-stimulating activity of LP2 will be further investigated. Other antigens will be administered in combination with LP2 to investigate whether LP2 also could enhance their ability to confer protection against *H. pylori*. For the improvement of antigen specific immune response, co-delivery system that delivers both the antigen and LP2 will be designed in the following studies.

## Data Availability

Publicly available datasets were analyzed in this study. This data can be found here: https://www.mrc-lmb.cam.ac.uk/genomes/dolop.

## Ethics Statement

This study was carried out in accordance with the recommendations of the Animal Ethical and Experimental Committee of the Third Military Medical University. The protocol was approved by the Animal Ethical and Experimental Committee of the Third Military Medical University.

## Author Contributions

H-BL and Q-MZ designed experiments. R-YX, M-FG, LG, CL, SL, LJ, LN, C-JM, and D-QC carried out experiments. H-BL, JL, ZJ, and SS analyzed experimental results. H-BL, Q-MZ, R-YX, and MG wrote the manuscript.

### Conflict of Interest Statement

The authors declare that the research was conducted in the absence of any commercial or financial relationships that could be construed as a potential conflict of interest.
