# Supplementary material for: Synthetic Lipopeptide Enhances Protective Immunity Against Helicobacter pylori Infection
Source: Front Immunol. 2019 Jun 14;10:1372. doi: 10.3389/fimmu.2019.01372 (PMC6587705; doi:10.3389/fimmu.2019.01372)
Supplement: Supplementary file 1 [file Data_Sheet_1.docx]

**Synthetic Lipopeptide Enhances Protective Immunity against *Helicobacter pylori* Infection**

Ruo-Yi Xue^1^, Mu-fei Guo^2^, Ling Guo^3^, Chang Liu^1^, Sun Li^1^, Jiao Luo^1^, Li Nie^1^, Lu Ji^1^, Cong-Jia Ma^1^, Da-Qun Chen^1^, Quan-Ming Zou^1*^, Hai-Bo Li^1*^

^1^ National Engineering Research Center of Immunological Products, Department of Microbiology and Biochemical Pharmacy, College of Pharmacy, Third Military Medical University, Chongqing 400038, P.R. China.

^2^ Chongqing Nankai Secondary School, Chongqing 400030, P.R. China

^3^ Chongqing Technical Center for Drug Evaluation and Certification, Chongqing 400014, P.R. China

Correspondence to Prof. Hai-Bo Li,

College of Pharmacy, Third Military Medical University, Chongqing 400038, P.R. China

E-mail address: lihaibo@tmmu.edu.cn (H.B. Li)

Or Prof. Quan-Ming Zou,

College of Pharmacy, Third Military Medical University, Chongqing 400038, P.R. China

E-mail address: qmzou2007@163.com (Q.M. Zou)

**Supplementary figures**


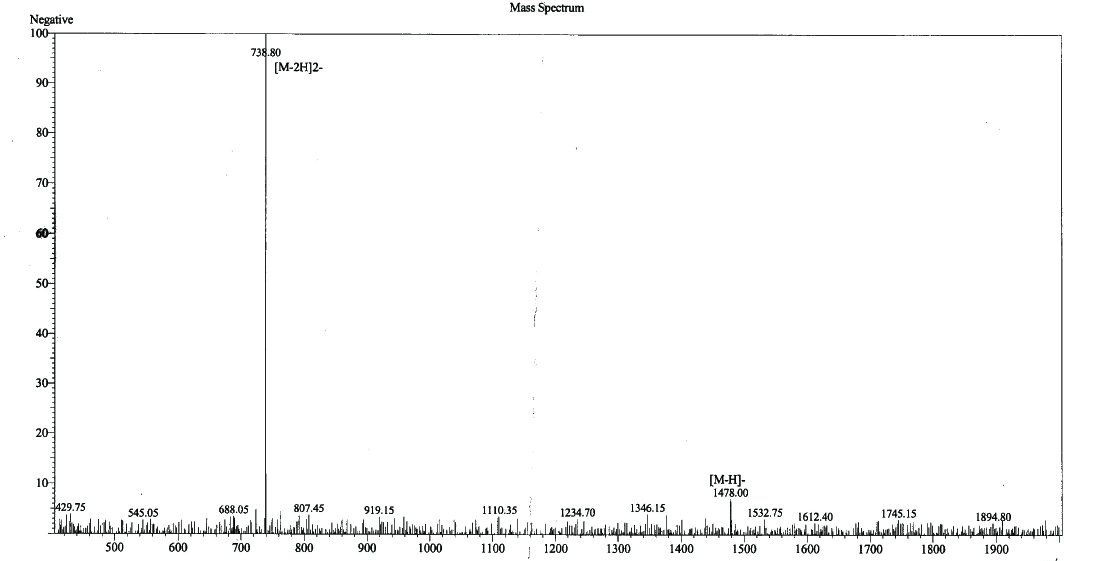


**Fig S1.** The mass spectrum of the synthetic lipopeptede LP1


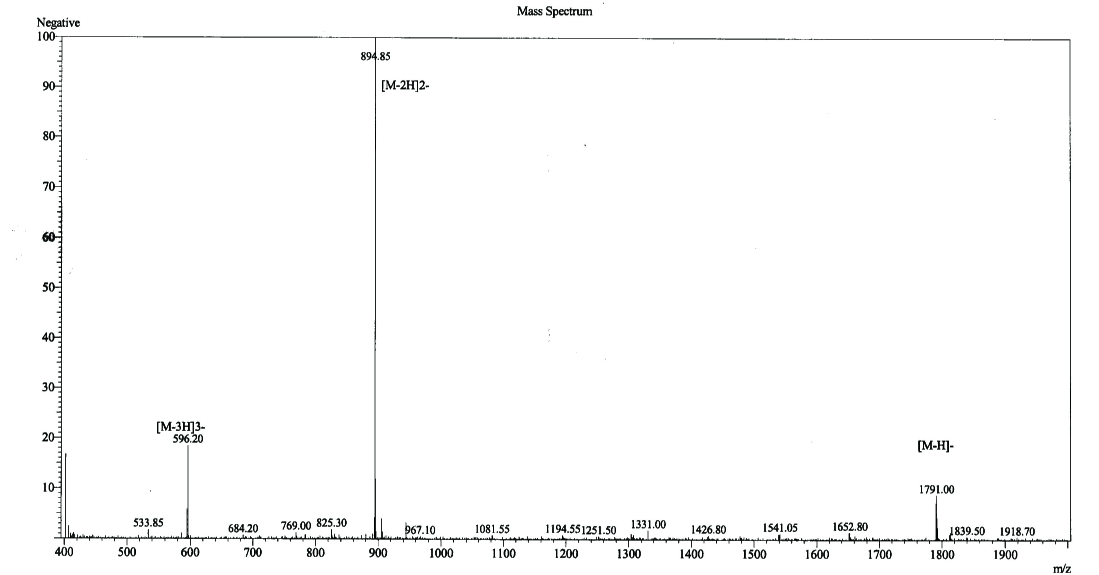


**Fig S2.** The mass spectrum of the synthetic lipopeptede LP2


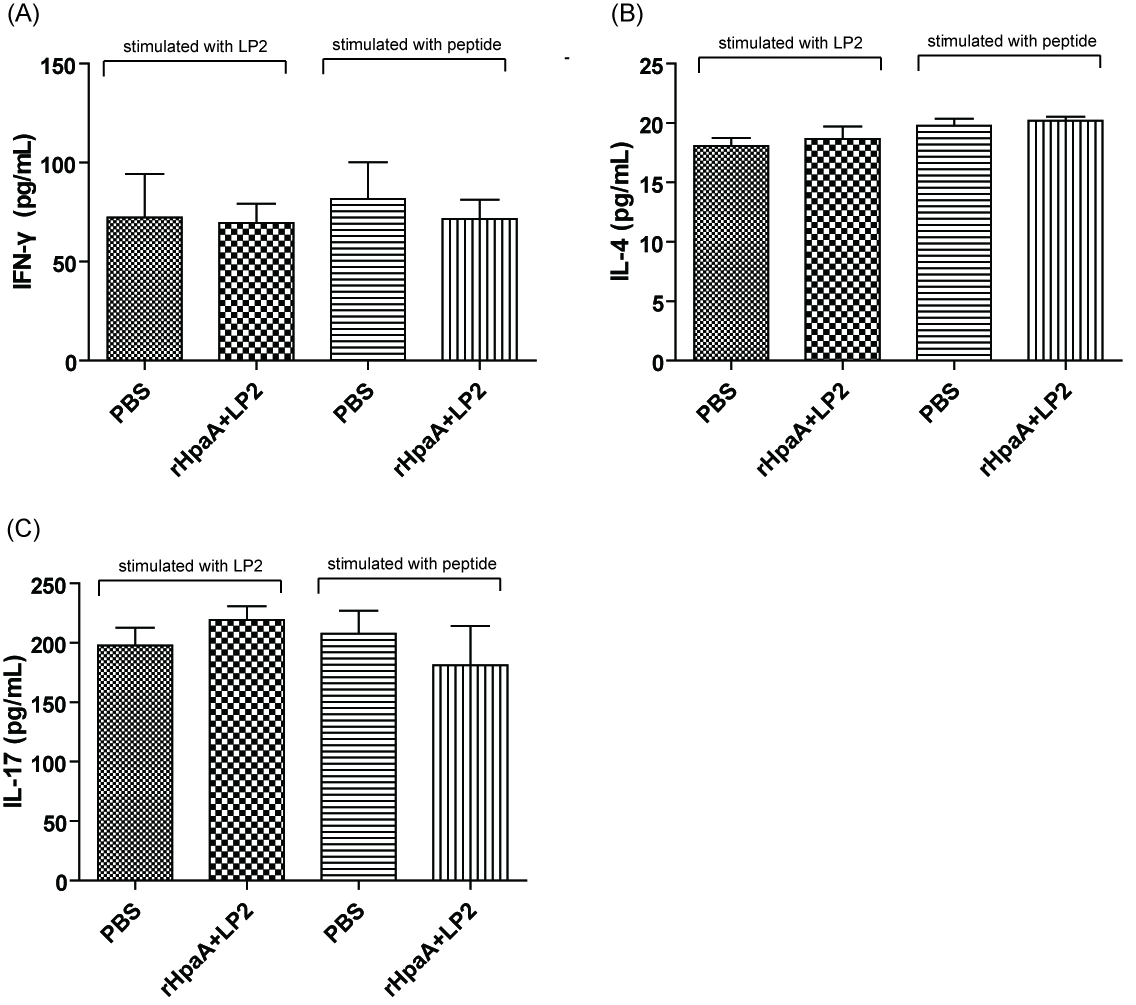


**Fig S3.** Cytokines production after stimulation of splenic lymphocytes with LP2 or the peptide. Mice were intranasally immunized with rHpaA plus LP2. Six week after final immunization, splenic lymphocytes from vaccinated group were isolated and stimulated with 10 μg/mL LP2 or the peptide for 48h. ELISA assays were used to measure the accumulation of IFN-γ (A), IL-4 (B), and IL-17 (C) in the supernatants of culture. Data are expressed as mean±S.D., n=5.
